# Effects of periodic mechanical stress on cytoskeleton dependent lipid raft-induced integrin ɑ1 activation in rat nucleus pulposus cells

**DOI:** 10.1007/s10735-023-10112-1

**Published:** 2023-01-31

**Authors:** Gongming Gao, Kewei Ren, Liang Chen, Xinru Li, Zitong Li, Yang Liu, Chenxi Ouyang, Hao Wang, Luming Nong, Hua Xie

**Affiliations:** 1 Department of Orthopedics, The Affiliated Changzhou No.2 People’s Hospital of Nanjing Medical University, 213000 Changzhou, China; 2 Department of Orthopedics, The Affiliated Jiangyin Hospital of Southeast University Medical School, Jiangyin, China; 3 Department of Orthopedics, First Affiliated Hospital of Soochow University, Suzhou, China; 4 Department of Orthopedics, Jintan People’s Hospital Affiliated to Jiangsu University, 213200 Jintan, China

**Keywords:** Periodic mechanical stress, Nucleus pulposus cells, Cytoskeleton, Lipid rafts, Integrin ɑ1

## Abstract

Extracellular matrix (ECM) production and nucleus pulposus (NP) cell migration increase under periodic mechanical stress (PMS), but the underpinning regulatory mechanism remains unclear. This work aimed to examine the regulatory effects of cytoskeleton-lipid raft-integrin α1 signaling in NP cells exposed to PMS. Briefly, In NP cells, cytoskeleton rearrangement, lipid raft aggregation and integrin α1 expression in the stress and control groups were assessed by immunofluorescent staining and immunoblot. In addition, cell migration and ECM gene expression were detected by a scratch test and quantitative reverse transcription polymerase chain reaction (qRT‑PCR), respectively. As a result, PMS up-regulated ECM gene expression and enhanced NP cell migration (both P < 0.05), accompanied by increased integrin α1, lipid raft, caveolin-3, F-actin and β-tubulin amounts. Pretreatment with the lipid raft inhibitor methyl-β-cyclodextrin (MβCD) or small interfering RNA (siRNA) targeting caveolin-3 resulted in decreased ECM mRNA synthesis and cell migration induced by PMS (both P < 0.05); meanwhile, integrin α1 expression was also reduced. F-actin and β-tubulin inhibition by cytochalasin D and colchicine, respectively, not only reduced ECM mRNA synthesis and cell migration (both P < 0.05), but also disrupted lipid raft and caveolin-3 amount increases induced by PMS in NP cells. In conclusion, PMS promotes ECM mRNA up-regulation and cell migration through the cytoskeleton-lipid raft-integrin α1 signaling pathway, inhibiting cytoskeleton and lipid rafts could block the cellular effects.

**Supplementary information:**

The online version contains supplementary material available at 10.1007/s10735-023-10112-1.

## Introduction

Disc degeneration disease(DDD)is a common ailment in the spine surgery department. Although the pathogenesis of DDD remains unclear, mechanical stress plays an important role in this process. In vivo, the physiological stress in the intervertebral disc varies with the different posture of the body (Wilke, 1999), which will affect the development, regeneration and degeneration of disc (Kroeber, 2005; Guehring, 2006). In vitro, it is known that mechanical stress can regulate NP cell bioactivities (Kuo, 2014; Feng, 2018; Kim, 2009). On the one hand, appropriate periodic mechanical stress (PMS) can promote NP cell proliferation and extracellular matrix (ECM) synthesis (Gao, 2018; Gao, 2016a; Gao, 2016b). On the other hand, the abnormal stress environment is one of the most important factors leading to NP cell degeneration (Kuo, 2014). However, the process in which mechanical stress signals are converted into chemical ones in NP cells needs further investigation.

Integrins constitute an important protein family on the surface of the cell membrane, which mediate cell adhesion, migration and other biological functions (Wu, 2019). Our previous study (Gao, 2016a) demonstrated that integrin α1 regulates ECM mRNA synthesis and NP cell migration under PMS. Integrin α1 can regulate the downstream phospholipase Cγ1 to convert mechanical signals into biochemical ones, but it remains unclear which protein regulates the pathway upstream of integrin α1.

Lipid rafts were firstly reported by researchers in 1997 (Simons, 1997) and systematically reviewed in 2002 (van, 2002). They constitute a heterogeneous structure located in the cell membrane and composed of cholesterol and sphingomyelin (Zajchowski, 2002). Most proteins in lipid rafts are associated with signal transduction (Katagiri, 2001; Harder 2004). The special structure of lipid rafts is helpful for cell signal transmission. For example, various proteins gather in lipid rafts, which will facilitate their interactions. It has been confirmed that lipid rafts play an important role in the stimulation feedback of different cells (such as lymphocytes (Verstraeten, 2010) and endothelial cells (Zeng, 2013) to mechanical stress, which is related to the structure and localization of lipid rafts on plasma membrane. But there is still a lack of relevant researches on the mechanism of lipid rafts participating in regulation of nucleus pulposus cells under the stress environment.

The cytoskeleton is a protein-fiber network structure in eukaryotes (Wang, 2020); it not only has critical functions in maintaining cellular morphology, external stress bearing, and maintaining the cell’s internal structure, but also contributes to regulating the metabolic function of cells. Besides, the cytoskeleton has been documented to interact with lipid rafts to affect cell signaling and function (Head, 2014).

In this study, we investigated the changes of integrin α1, lipid rafts and cytoskeleton in NP cells exposed to PMS as well as the associated regulatory mechanisms.

## Materials and methods

### Nucleus pulposus (NP) cell culture

NP cells from male Sprague-Dawley rats (4-week-old) were isolated and cultured as described in a previous study (Gao, 2016a). Briefly, the nucleus pulposus from rat thoracic and lumbar spines were collected and digested in 1.5% type II collagenase (Gibco; Thermo Fisher Scientific, Inc., USA) to get primary cells.Experiments involving animals had approval from the Ethics Committee of Nanjing Medical University. NP cells were passaged at approximately 80% confluence. Passage 2 NP cells at a density of 10^5^/ml were seeded on slides (25 mm × 25 mm), and assessed in subsequent experiments.

### Periodical mechanical stress equipment

A perfusion culture system with periodic stress field was designed by our team and produced by Taixing Experimental Instrument Factory (China). In short, this system consisted of a reciprocating intensifier pump and an air-tight cell culture device. It provides PMS in the range of 0-0.3 MPa at 0–1 Hz. PMS at 0-200 kPa (0.1 Hz) was selected for the experiments according to our previous studies (Gao, 2016b, Liang, 2017).

### Experimental grouping

The experiments were carried out in two steps. First, the cells were assigned to the control and stress groups. The stress group underwent PMS, while control cells were not subjected to stress. The time of stress treatment depended on the different experiments. Briefly, 24 h stress treatment was performed for cell migration assay, and 8 h stress treatment was used for quantitative reverse transcription polymerase chain reaction (qRT‑PCR)and immunoblot. Then, cell migration assay, qRT‑PCR, lipid raft aggregation, cytoskeleton rearrangement and integrin α1 expression were detected in different groups. Secondly, the cells were pretreated with small interfering RNA (siRNA) targeting caveolin-3, methyl-β-cyclodextrin (MβCD), colchicine and cytochalasin D, respectively, before exposure to PMS. Then, the abovementioned assays were repeated.

### Disruption of lipid rafts and the cytoskeleton

MβCD (C4555, Sigma-Aldrich, USA) is known as an inhibitor that disrupts the accumulation of lipid rafts (Deng, 2008). In order to investigate the biological effects of disrupted lipid rafts under stress environment, cells were pretreated with 20 mM MβCD for 60 min before the subsequent assays. Cytochalasin D (C2618, Sigma-Aldrich) and colchicine (C3915, Sigma-Aldrich) are F-actin and β-tubulin inhibitors, respectively. In order to assess the biological effects of F-actin or β-tubulin disruption under stress environment, cells were pretreated with 3µM cytochalasin D or 20µM colchicine for 90 min before the subsequent assays.

### Cell transfection

The siRNA sequences were manufactured by Shanghai GenePharma (China): caveolin-3 siRNA 1, forward 5’- GUGAGCUACACCACUUUCATT − 3’ and reverse 5’- UGAAAGUGGUGUAGCUCACTT − 3’; caveolin-3 siRNA 2, forward 5’- GCUACCUGAUUGAGAUCCATT − 3’ and reverse 5’- UGGAUCUCAAUCAGGUAGCTT − 3’; caveolin-3 siRNA 3, forward 5’- GCAACAUUAAGGUGGUGCUTT − 3’ and reverse 5’- AGCACCACCUUAAUGUUGCTT − 3’; negative control (NC) siRNA, forward 5’- UUCUCCGAACGUGUCACGUTT-3’ and reverse 5’- ACGUGACACGUUCGGAGAATT-3’. For transfection, siRNA (75 pM) was mixed with Lipofectamine 2000 (Invitrogen, USA) in 50 µl Opti‑MEM medium (Gibco; USA) as directed by both manufacturers. Then, slides were incubated for 6 h before the subsequent experiments.

### Immunoblot

Immunoblot was carried out as described in a previous report (Gao, 2016b). In brief, following treatment with RIPA lysis buffer (P0013, Beyotime, China), total protein was quantitated with a BCA protein assay kit (P0012, Beyotime).

After separation by sodium dodecyl sulfate polyacrylamide gel electrophoresis (SDS-PAGE), the protein bands underwent transfer onto polyvinylidene fluoride (PVDF) membranes. This was followed by overnight incubation (4℃) with primary antibodies raised against integrin α1 (1:1,000; sc-271,034, Santa Cruz, USA), caveolin-1 (1:1,000; sc-894, Santa Cruz), caveolin-2 (1:1,000; ab2911; Abcam, UK), caveolin-3 (1:1,000; ab2912; Abcam), and GAPDH (1:5,000; AP0063; Bioworld Technology, USA). Following washing with PBS, goat anti-mouse (GAM007; MultiSciences Technology, USA) or anti-rabbit (GAR0072; MultiSciences Technology) IgG-HRP was added for 1 h at ambient. Finally, Immobilon™ Western Chemiluminescent HRP substrate reagent (EMD Millipore, USA) was employed for development. Immunoreactive bands were captured and assessed on a Bio-Rad Gel Doc Imaging System (Bio-Rad Laboratories, USA).

### Quantitative reverse transcription polymerase chain reaction (qRT‑PCR)

Total RNA from rat NP cells was extracted with TRIzol regent (Invitrogen) and reverse transcribed with a PrimeScript RT Master Mix kit (RR036, Takara, Japan). Then, qRT‑PCR was carried out with a SYBR Premix Ex Taq II kit (RR820, Takara) on a Step One Plus Real-Time PCR system. Primers were: aggrecan, forward 5’-CCCTACCCTTGCTTCTCCA-3’ and reverse 5’-CTTGAGAGGCACTCATCAATGT-3’; Col2A, forward 5’-GACCCCCAGGTTCTAATGG-3’ and reverse 5’GCACCTTTGGGACCATCTT-3’; β‑actin, forward 5’-GCAGAAGGAGATTACTGCCCT-3’ and reverse 5’-GCTGATCCACATCTGCTGGAA-3’.

### Cellular immunofluorescent staining

Cell samples underwent treatment with 5 µg/ml of cholera toxin B-fluorescein isothiocyanate (CTB-FITC; c34775; Thermo Fisher Scientific, USA) at room temperature for 30 min. Then, cells were permeabilized using 0.1% Triton X-100 after fixation with 4% paraformaldehyde. This was followed by successive incubations with primary (4 °C, overnight) and secondary (4 °C, 2 h in the dark) antibodies. The primary antibodies were raised against β-Tubulin (1:500; ab009, MultiSciences Technology), F-actin (1:500; ab205; Abcam) and caveolin-3 (1:1,000; ab2912; Abcam). The secondary antibodies were as follows: Donkey anti-rabbit IgG-Alexa Fluor® 647 (A31573; Thermo Fisher Scientific) and Goat anti-mouse IgG-Alexa Fluor® 555 (A21424; Thermo Fisher Scientific). Finally, DAPI (D9542; Sigma-Aldrich) was used for counterstaining at ambient for 20 min before observation under an A1 laser confocal microscope (Nikon, Japan).

### Cell migration assay

The migratory capacity of NP cells was evaluated by a scratch test as previously described (Gao, 2016a). Briefly, the scratch test was performed when cells on slides were about 80% confluent. Following treatment, a 200 µl pipette tip was used to produce scratches. Then, the cells were cultured in an incubator with serum-free medium at 37˚C, 5% CO_2_ for 24 h. After observation under a microscope (Olympus, Japan), further analysis was performed with ImageJ 1.43 (imagej.nih.gov).

### Statistical analysis

Data are mean ± standard deviation (SD), and GraphPad Prism (GraphPad Software, USA) was utilized for analysis. Unpaired t-test was performed to compare group pairs. Multiple group comparisons were performed by one-way ANOVA followed by Dunnett’s multiple comparisons. P < 0.05 indicated statistical significance.

## Results

### PMS promotes ECM mRNA up-regulation, cell migration and integrin α1 upregulation

**Fig. 1:**
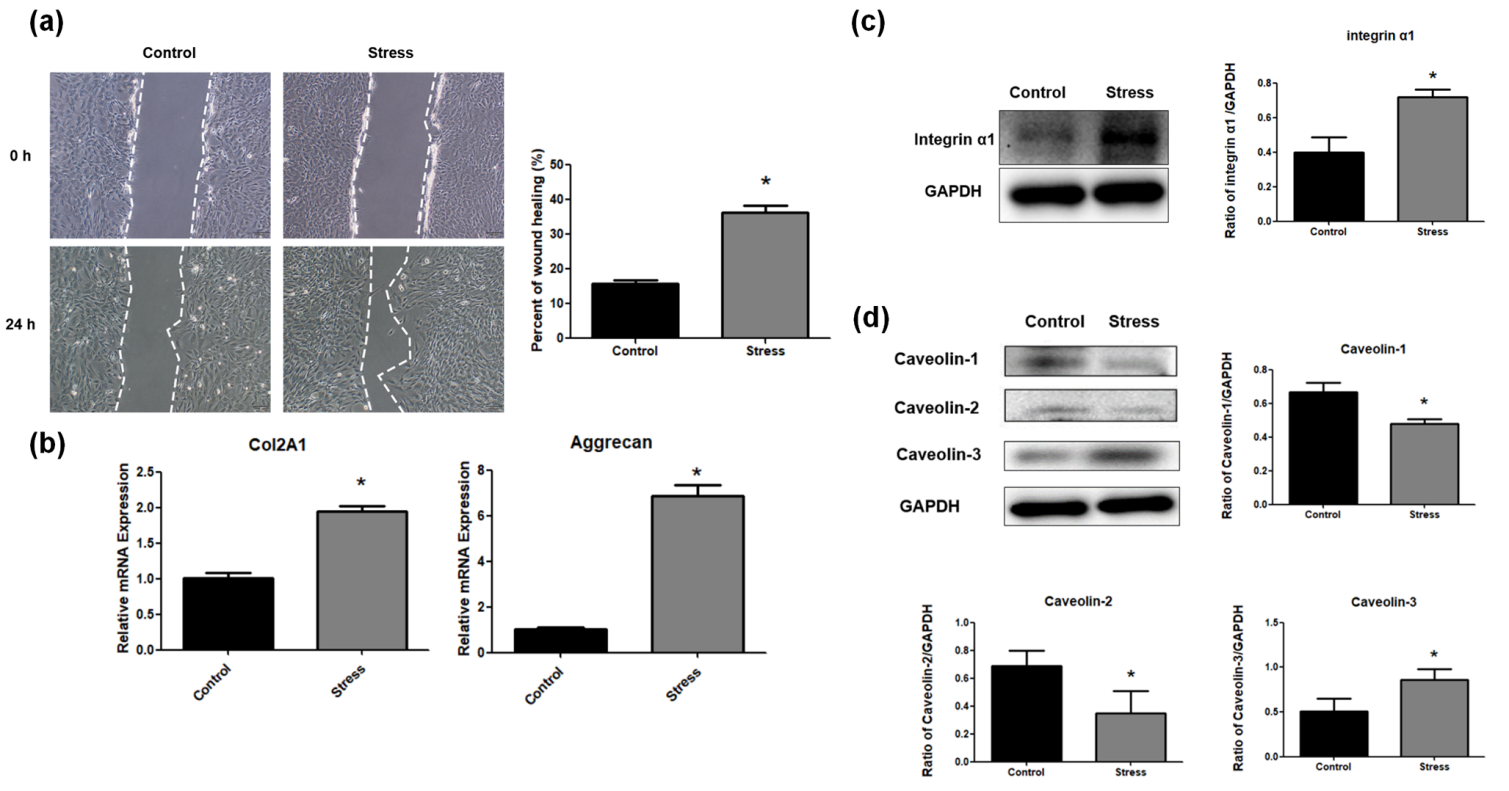
Promotion effects of PMS on ECM production, cell migration and integrin α1 expression. (a) NP cell migration was evaluated by a scratch assay. The difference between scratch width at 0 and 24 h divided by scratch width at 0 h was equal to the percent of wound healing (Scale bar=100 μm). Data were expressed as mean ± standard deviation (SD) (n=5). *P<0.05, compared with the control group. (b) RT-qPCR analysis for mRNA expression of Col2A1 and aggrecan after 8 h stress treatment. Data were expressed as mean ± SD. *P<0.05, compared with the control group. (c) Integrin α1 protein expression was detected by Western blot after 8 h stress treatment. (d) The results of Western blot for caveolin-1, caveolin-2 and caveolin-3 in control and 8 h stress group

As reported previously by our team (Gao, 2016a), in comparison with control cells, PMS (0.2 MPa; 0.1 Hz; 24 h) markedly enhanced cell migration (P < 0.05, Fig. 1a). PMS (0.2 MPa; 0.1 Hz; 8 h) upregulated ECM-associated Col2A1 and aggrecan (P < 0.05, Fig. 1b), and upregulated integrin α1 (Fig. 1c).

### PMS promotes lipid raft aggregation and caveolin-3 upregulation in NP cells

**Fig. 2:**
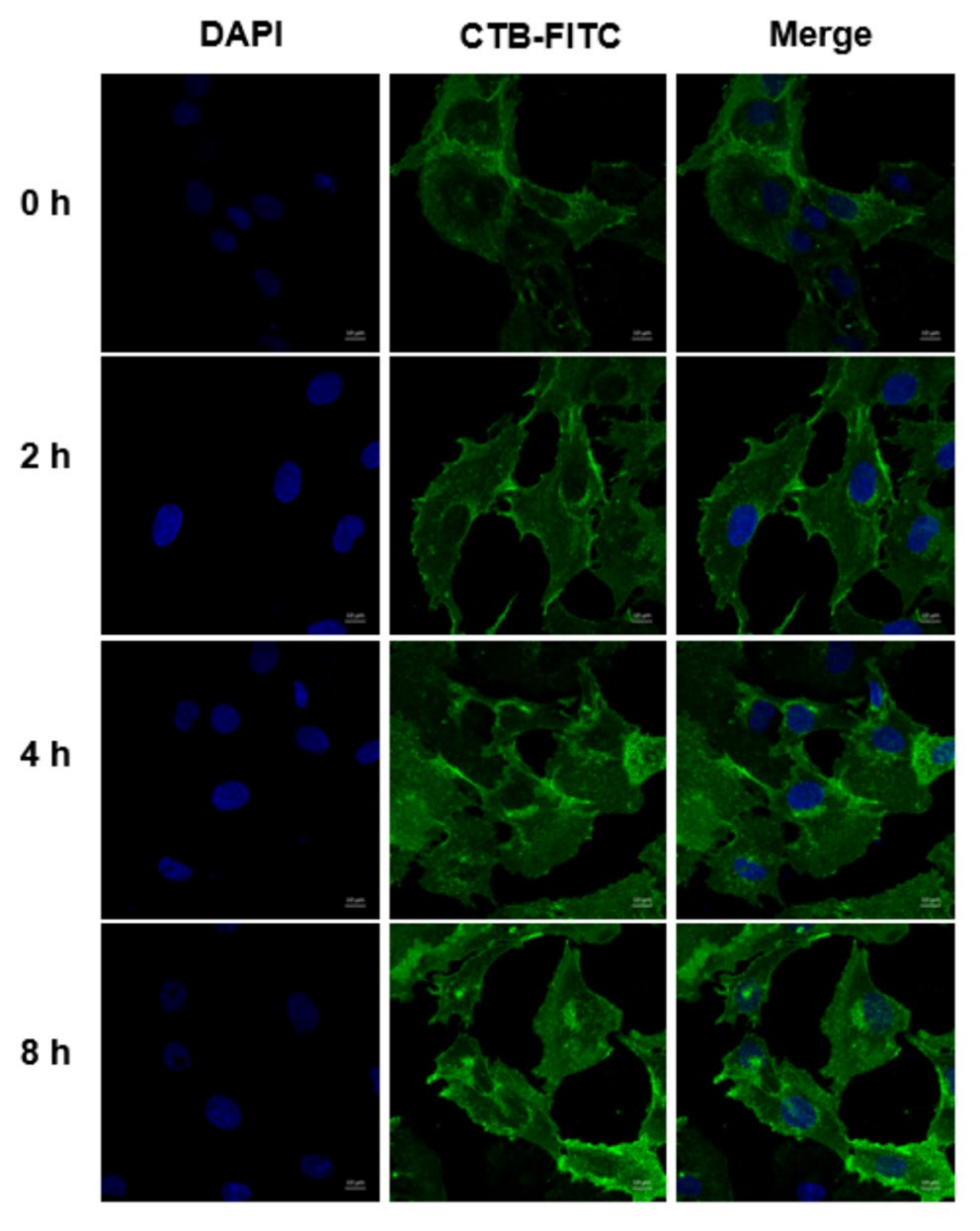
Promotion effects of PMS on lipid raft aggregation. CTB-FITC staining for observing lipid raft aggregation at 0 h, 2 h, 4 and 8 h time-point after stress treatment (Scale bar=10 μm). The accumulation of lipid rafts was the most significant after 8 h of stress treatment

CTB-FITC staining reflects lipid raft aggregation. In comparison with control cells, lipid raft aggregation was obvious at different time-points (2 h, 4 and 8 h) of stress treatment, especially in the 8 h stress treatment group (Fig. 2). Then, 8 h stress treatment was selected for subsequent assays. It was found that 8 h stress treatment upregulated caveolin-3, but not caveolin-1 or caveolin-2 by cellular western blot (Fig. 1d).

### Lipid rafts and caveolin-3 are required for PMS-induced ECM mRNA up-regulation, cell migration and integrin α1 upregulation

**Fig. 3:**
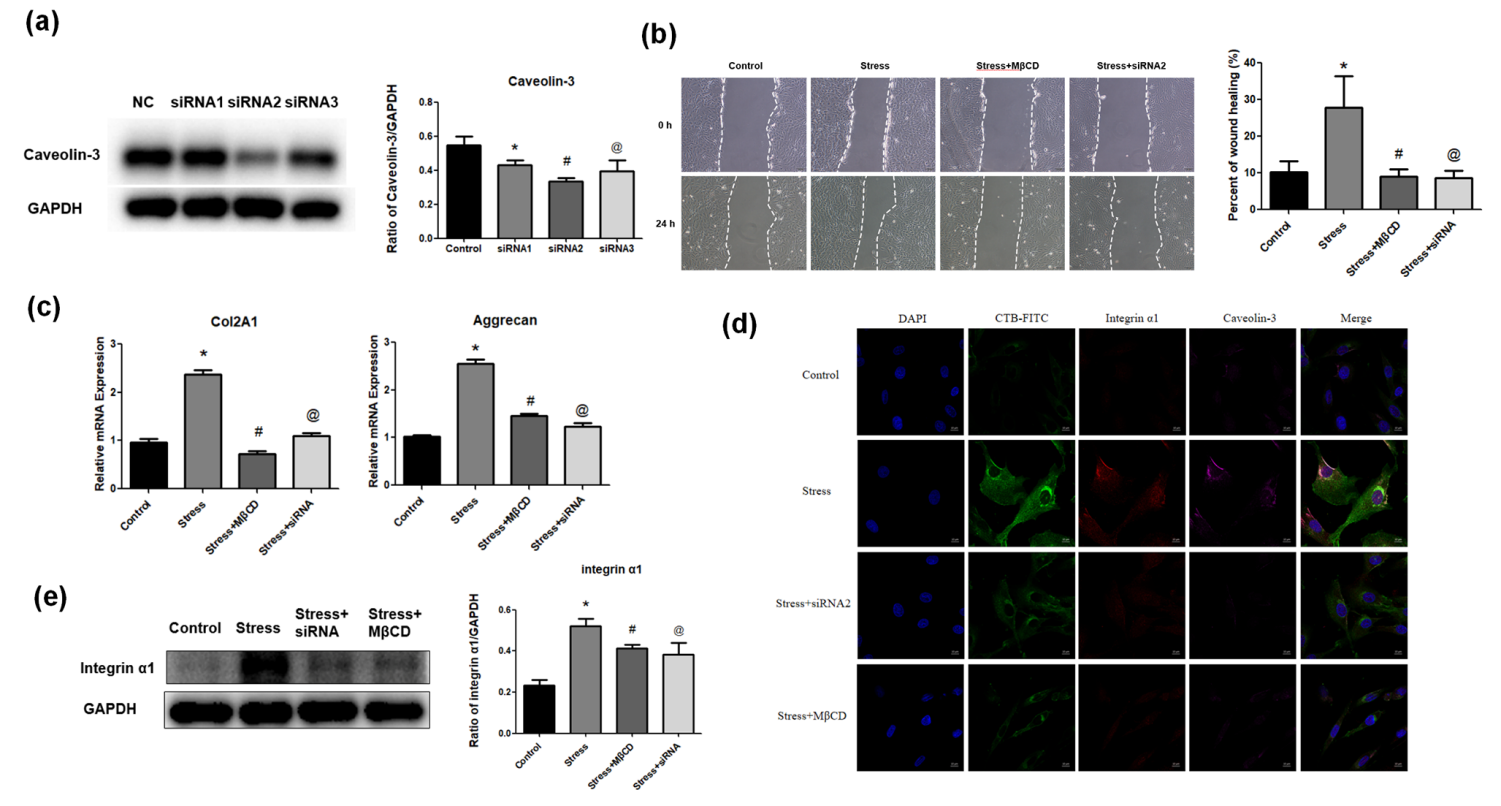
Role of lipid rafts and caveolin-3 in PMS-induced ECM production, cell migration and integrin α1 upregulation. (a) Western blot for caveolin-3 protein expression after blocking via siRNA. (b) NP cell migration was evaluated by a scratch assay after 24 h stress treatment. Representative images were showed in the control group, stress group, stress + MβCD group and stress + siRNA group. Data were expressed as mean ± SD (n = 5). *P<0.05, compared with the control group. #P<0.05, compared with the stress group. @P<0.05, compared with the stress group. (c) After 8 h stress treatment, RT-qPCR analysis for mRNA expression of Col2A1 and aggrecan in the control group, stress group, stress + MβCD group and stress + siRNA group. *P<0.05, compared with the control group. #P<0.05, compared with the stress group. @P<0.05, compared with the stress group. (d) After 8 h stress treatment, cellular immunofluorescent staining for lipid rafts, caveolin-3 and integrin α1 expression in the control group, stress group, stress + MβCD group and stress + siRNA group (Scale bar=10 μm). (e) After 8 h stress treatment, Western blot for integrin α1 protein expression in the control group, stress group, stress + MβCD group and stress + siRNA group

In order to further explore the regulatory mechanism of lipid rafts and caveolin-3 under stress environment, the siRNA technology and MβCD were employed to inhibit caveolin-3 and lipid rafts, respectively. First, siRNA sequences (siRNA 2) with the best inhibitory effects on caveolin-3 (Fig. 3a) were selected. After pretreatment of NP cells with siRNA 2 and MβCD, respectively, inhibition of lipid rafts and caveolin-3 significantly reduced cell migration (P < 0.05, Fig. 3b) and ECM mRNA production (P < 0.05, Fig. 3c) in comparison with control cells under PMS. We further assessed the associations of lipid rafts, caveolin-3 and integrin α1. Both cellular immunofluorescent staining (Fig. 3d) and Western blot (Fig. 3e) demonstrated that inhibition of lipid rafts and caveolin-3, respectively, suppressed PMS-induced integrin α1 upregulation.

### PMS increases F-actin and β-tubulin amounts

**Fig. 4:**
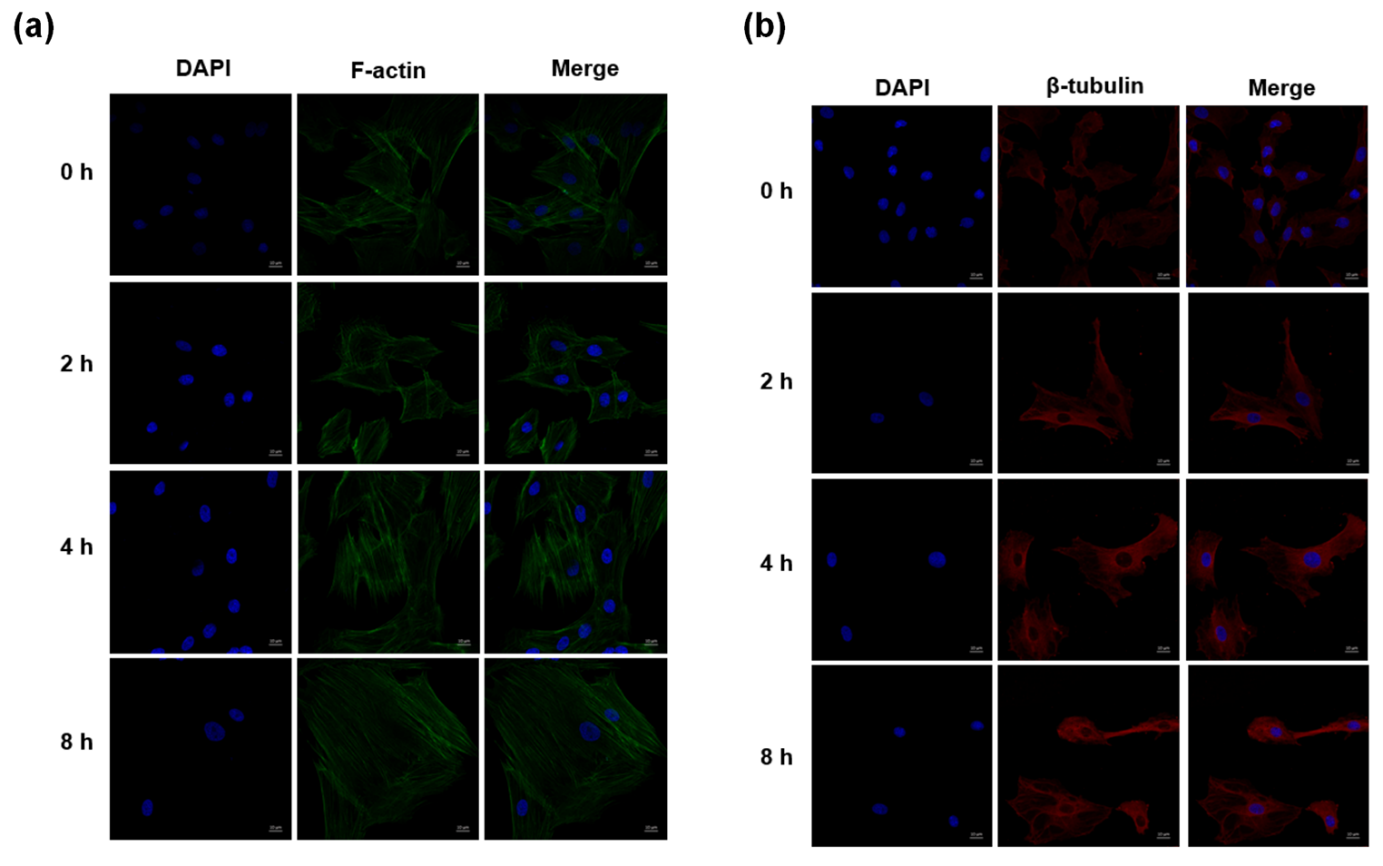
Cellular immunofluorescent staining for F-actin and β-tubulin expression. (a) Cellular immunofluorescent staining for observing F-actin expression at 0 h, 2 h, 4 and 8 h time-point after stress treatment (Scale bar=10 μm). (b) Cellular immunofluorescent staining for observing β-tubulin expression at 0 h, 2 h, 4 and 8 h time-point after stress treatment (Scale bar=10 μm)

Cellular immunofluorescent staining showed that F-actin and β-tubulin amounts were obviously increased after 2 h, 4 and 8 h of stress treatment in comparison with control cells (Fig. 4a and b).

### F-actin and β-tubulin are required for PMS induced increase of lipid rafts and caveolin-3 upregulation in NP cells

**Fig. 5:**
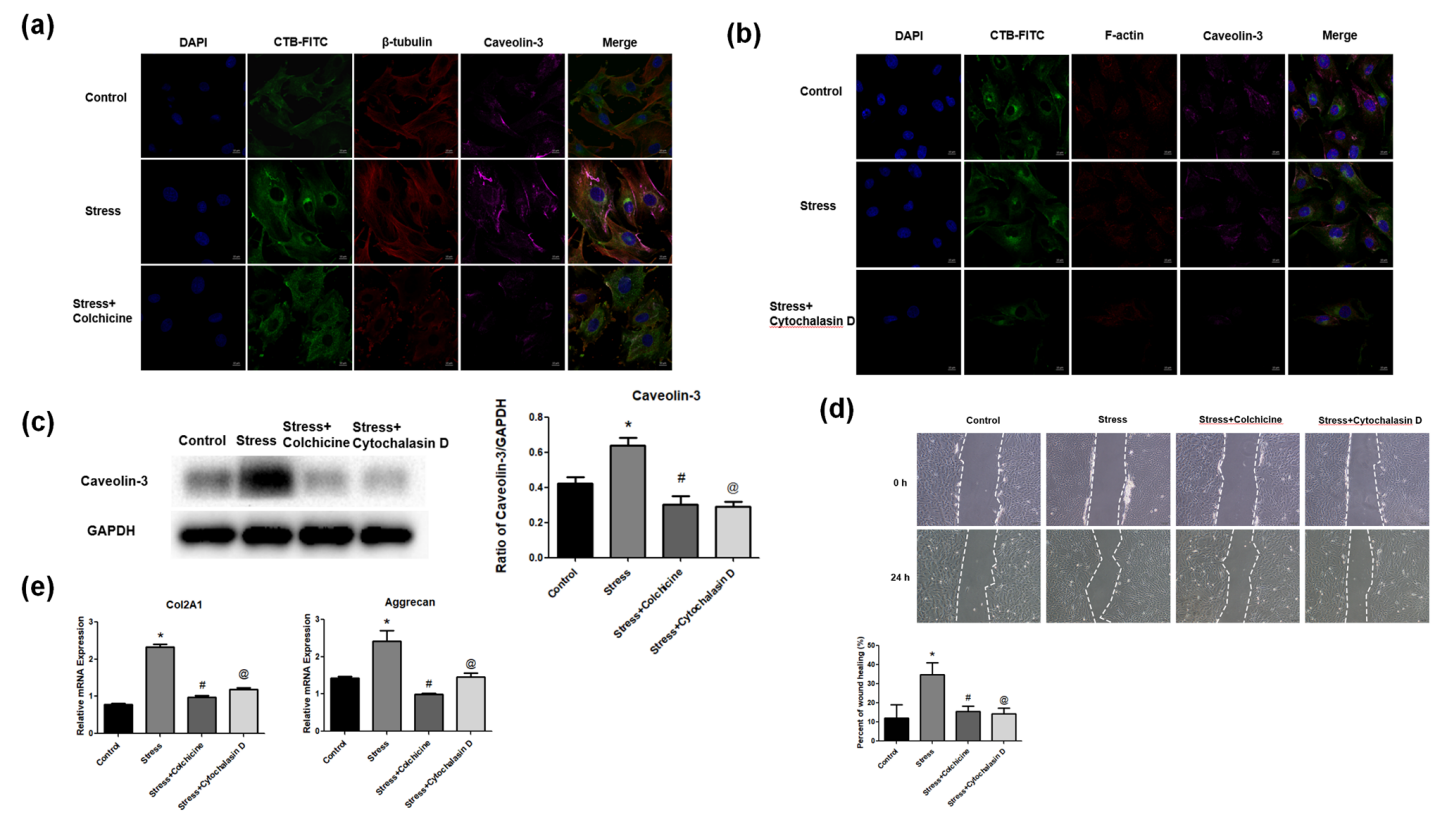
Role of F-actin and β-tubulin in PMS‑induced increase of lipid rafts and caveolin-3 upregulation in NP cells. (a) After 8 h stress treatment, cellular immunofluorescent staining for lipid rafts, β-Tubulin and caveolin-3 expression in the control group, stress group and stress + Colchicine group (Scale bar=10 μm). (b) After 8 h stress treatment, cellular immunofluorescent staining for lipid rafts, F-actin and caveolin-3 expression in the control group, stress group and stress + Cytochalasin D group (Scale bar=10 μm). (c) After 8 h stress treatment, Western blot for caveolin-3 protein expression in the control group, stress group, stress + Colchicine group and stress + Cytochalasin D group. (d) NP cell migration was evaluated by a scratch assay after 24 h stress treatment. Representative images were showed in the control group, stress group, stress + Colchicine group and stress + Cytochalasin D group. Data were expressed as mean ± SD (n = 5). *P<0.05, compared with the control group. #P<0.05, compared with the stress group. @P<0.05, compared with the stress group. (e) After 8 h stress treatment, RT-qPCR analysis for mRNA expression of Col2A1 and aggrecan in the control group, stress group, stress + Colchicine group and stress + Cytochalasin D group. Data were expressed as mean ± SD. *P<0.05, compared with the control group. #P<0.05, compared with the stress group. @P<0.05, compared with the stress group

To further investigate the regulatory relationships among cytoskeleton, lipid rafts and caveolin-3, the amounts of lipid rafts and caveolin-3 were examined after pretreatment of NP cells with F-actin (cytochalasin D) and β-tubulin (colchicine) inhibitors, respectively. Figure 5a shows that in comparison with control cells, PMS increased the amounts of lipid rafts, caveolin-3 and β-Tubulin, which were reduced after treatment with colchicine. Similarly, in comparison with control cells, stress treatment elevated the amounts of lipid rafts, caveolin-3 and F-actin, which were also decreased by treatment with cytochalasin D (Fig. 5b). After pretreatment of NP cells with colchicine and cytochalasin D, respectively, inhibition of β-tubulin and F-actin could significantly reduce cell migration (P < 0.05, Fig. 5d) and ECM mRNA level (P < 0.05, Fig. 5e) in comparison with control cells under PMS.

## Discussion

This study assessed the transduction of mechanical signals to biochemical ones in NP cells. The results showed that the cytoskeleton, lipid rafts (especially the lipid raft component caveolin-3) and integrin α1 participate in mechanical-biochemical signal transmission in NP cells.

Integrin subunits α1, α5 and α6 have been detected in intervertebral disc tissues (Nettles, 2004). Some integrins are important in regulating the attachment of NP cells to laminins (Bridgen, 2013) as well as NP cell survival in hypoxia (Risbud, 2005). Our previous report demonstrated that integrin α1 is critical in regulating migration and ECM mRNA production in NP cells under stress environment (Gao, 2016a). In this study, corroborating our previous findings, PMS upregulated integrin α1 in NP cells in a lipid raft dependent manner. Our results showed that integrin α1 was significantly downregulated after MβCD-mediated destruction of lipid rafts (Dykstra, 2003) in NP cells. Relevant evidences confirm that lipid rafts and integrin interact to regulate cell migration, regeneration and proliferation (Bi, 2018, Antelmi, 2013; Lee, 2016). However, in the process of mechanical-biochemical signal transmission, we firstly elucidated the relationship between lipid rafts and integrin α1.

In this study, CTB-FITC was used to label lipid rafts. CTB directly binds to GM1 gangliosides in lipid rafts, promoting lipid raft clustering in cells (Deng, 2008, Bi, 2001). The above results confirmed that compared with the control group, mechanical stress induced lipid raft aggregation significantly. Caveolae is the most important component of lipid rafts (Huo, 2003), while caveolin represents a marker protein of caveolae, with three variants including caveolin-1, caveolin-2 and caveolin-3 (Williams, 2004). Several studies have focused on caveolin-1 in the intervertebral disc. For instance, it was reported that caveolin-1 is upregulated with disc degeneration (Bach, 2016) but not in association with age (Heathfield, 2008). Furthermore, caveolin‑1 participates in NP cell senescence after oxidative injury (Ding, 2017) and may constitute an ideal target for the treatment of IVD degeneration (Smolders, 2013). However, no report has assessed the role of caveolin-3 in NP cells. As shown above, caveolin-3 amounts were significantly elevated in comparison with those of caveolin-1and 2 under mechanical stress. After blocking the caveolin-3 protein, extracellular matrix amounts and NP cell migration were decreased significantly compared with the stress group, again showing that caveolin-3 has a critical function in signal regulation under stress stimulation.

Previous studies demonstrated that the cytoskeleton is critical in modulating metabolic function in NP cells. The cytoskeleton consists of three parts, including microtubules (MTs), microfilaments (MFs), and intermediate filaments (IFs) (Pegoraro, 2017). Microtubules are mainly composed of tubulin, while microfilaments are mostly made of actin. It is known that leptin can upregulate β-actin, F-actin and vimentin in NP cells, inducing cytoskeleton remodeling through the RhoA/ROCK pathway (Li, 2013; Li 2014). Li et al. demonstrated that cyclic tensile strain can stimulate F-actin reorganization in NP cells and upregulate type II collagen (Li, 2011). Similarly, our results showed that PMS promoted cytoskeleton (β-Tubulin and F-actin) rearrangement in NP cells. Based on these findings, we further assessed whether cytoskeleton rearrangement was related to lipid raft aggregation and caveolin-3 upregulation. Following treatment with cytochalasin D and colchicine to inhibit F-actin and β-tubulin, respectively, it was found that lipid raft aggregation and caveolin-3 upregulation in NP cells induced by stress were also significantly weakened. Although it is known that lipid raft aggregation depends on cytoskeleton activation (Badizadegan, 2004, Head, 2006), this is the first time to draw similar conclusions under mechanical stress stimulation.

In *vivo*, the development, degeneration and regeneration of the intervertebral disc are inseparable from the stress environment. Therefore, this study of the nucleus pulposus cell regulation mechanism under the stress environment is closer to reality and has more profound significance. We demonstrated that the Src-GIT1-ERK1/2 pathway (Gao, 2018) and integrin α1-PLCγ1 (Gao, 2016a) were involved in the regulation chain. Combined with this study, from cytoskeleton, cell membrane structure (lipid rafts) and transmembrane protein integrin α1 to transporters in cytoplasm, we have basically clarified the transmission of signal pathways under stress environment. However, the limitation of this study is that all data is based on monolayer cell culture system, the further research will be performed in the three-dimensional cell culture system in the future.

**Fig. 6:**
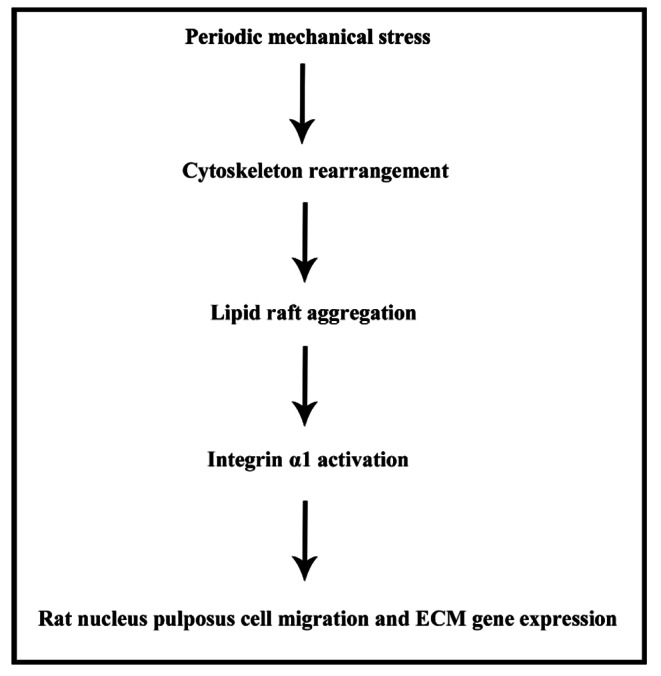
Schematic diagram depicts the signal regulation chain under stress environment

Overall, this work demonstrated that PMS could promote cytoskeleton rearrangement and induce lipid raft aggregation in NP cells, resulting in integrin α1 upregulation and increased ECM mRNA production and cell migration (Fig. 6). These results reveal the regulatory mechanism underlying the biological functions of NP cells under stress environment. These findings provide a theoretical basis for further DDD research.

## Electronic supplementary material

Below is the link to the electronic supplementary material.

Supplementary Material 1: The other two Western blot images for caveolin-3 protein expression after blocking via siRNA (Fig. 3a)

## Data Availability

The data supporting the findings of this study are available from the corresponding author upon reasonable requests.

## Abbreviations

DDD: Disc degeneration disease
PMS: periodic mechanical stress
ECM: extracellular matrix
NP: Nucleus pulposus
MβCD: methyl-β-cyclodextrin
CTB-FITC: cholera toxin B-fluorescein isothiocyanate
