# Supplementary figures and images for: Effects of periodic mechanical stress on cytoskeleton dependent lipid raft-induced integrin ɑ1 activation in rat nucleus pulposus cells
Source: J Mol Histol. 2023 Jan 31;54(1):67–75. doi: 10.1007/s10735-023-10112-1 (PMC9908706; doi:10.1007/s10735-023-10112-1)

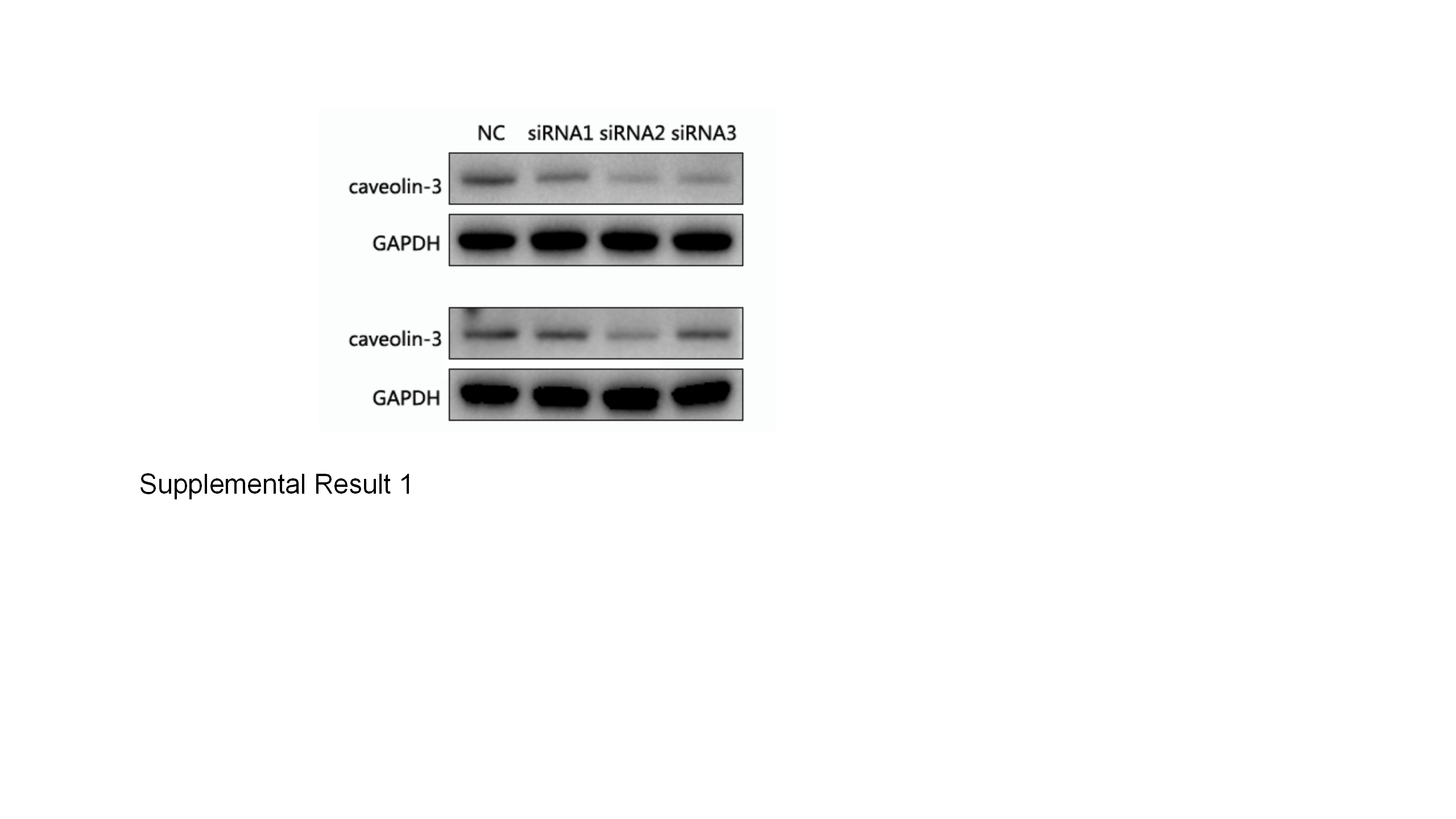

Supplement: Supplementary file 1 — Supplementary Material 1: The other two Western blot images for caveolin-3 protein expression after blocking via siRNA (Fig. 3a) [file 10735_2023_10112_MOESM1_ESM.tif]
